# Dr. O. F. Harris

**Published:** 1892-10

**Authors:** 


### Obituary.

### DR. O. F. HARRIS.

Whereas, The hand of Divine Providence having removed from our midst Dr. O. F. Harris, of Worcester, after a life of usefulness and honor, we place upon our records our appreciation of his wisdom and skill.

*Resolved*, That we tender to his family our sincere condolence and sympathy.

(Signed) D. B. Ingalls,
S. G. Stevens,
J. W. Bailey,
*Committee.*

(Attest) Edgar O. Kinsman,

*Secretary.*

